# Multifractality approach of a generalized Shannon index in financial time series

**DOI:** 10.1371/journal.pone.0303252

**Published:** 2024-06-21

**Authors:** Felipe S. Abril-Bermúdez, Juan E. Trinidad-Segovia, Miguel A. Sánchez-Granero, Carlos J. Quimbay-Herrera

**Affiliations:** 1 Department of Physics, Universidad Nacional de Colombia, Bogotá, D.C., Colombia; 2 Department of Economics and Business, Universidad de Almería, Almeria, Spain; 3 Department of Mathematics, Universidad de Almería, Almeria, Spain; URV: Universitat Rovira i Virgili, SPAIN

## Abstract

Multifractality is a concept that extends locally the usual ideas of fractality in a system. Nevertheless, the multifractal approaches used lack a multifractal dimension tied to an entropy index like the Shannon index. This paper introduces a generalized Shannon index (GSI) and demonstrates its application in understanding system fluctuations. To this end, traditional multifractality approaches are explained. Then, using the temporal Theil scaling and the diffusive trajectory algorithm, the GSI and its partition function are defined. Next, the multifractal exponent of the GSI is derived from the partition function, establishing a connection between the temporal Theil scaling exponent and the generalized Hurst exponent. Finally, this relationship is verified in a fractional Brownian motion and applied to financial time series. In fact, this leads us to proposing an approximation called local fractional Brownian motion approximation, where multifractal systems are viewed as a local superposition of distinct fractional Brownian motions with varying monofractal exponents. Also, we furnish an algorithm for identifying the optimal *q*-th moment of the probability distribution associated with an empirical time series to enhance the accuracy of generalized Hurst exponent estimation.

## 1 Introduction

The scaling property of a mathematical function is a crucial tool for understanding system variations based on exponents associated with universality classes [1]. In stochastic processes, this property is explored by examining the invariance of the probability distribution pattern of a random variable [2]. For stationary time series, the identification of a single exponent, denoted as *δ* and referred to as the scaling exponent, is found to be adequate. Early pioneers such as B. Mandelbrot and H. Stanley played significant roles in calculating this exponent in time series [1, 3–6]. Since the 1980s, various methods, including the precise Shannon entropy calculation in generated sub-series via the diffusive trajectory algorithm, have been proposed for determining the scaling exponent *δ* [7]. Notably, the diffusive trajectory algorithm generates multiple sub-series by adding consecutive terms, akin to a simple Brownian motion process, and computes the theoretical scaling exponent of a Lévy flight [7, 8]. However, challenges arise as the scaling exponent *δ* doesn’t always coincide with the Hurst exponent (*H*) which describes the evolution of the second moment around the origin in an anomalous diffusive process [9–12].

On the other hand, the Theil index (*T*) is an inequality measure devised by economist Henri Theil and formulated in terms of an entropy index [13]. Also, this index boasts a decomposition property wherein the overall income inequality of a population is derived from subgroup inequalities [14, 15]. Employed in econophysics, the Theil index has been used to explore correlations in time series, transforming them into entropy time series based on time window size [16]. Its applications extend to comprehending equilibrium states in free market models [17], analyzing regional changes in foreign aid distribution using an entropic approach [18], and studying income distribution in countries [19]. The Theil index also finds relevance in characterizing racial segregation, measuring redundancy in data, and assessing diversity [20–22].

While comparing Theil indices across populations of different sizes poses challenges, normalization by the logarithm of data number renders them comparable. Normalized, the Theil index mirrors the Gini index, introduced by sociologist Corrado Gini in 1912, with both reaching 0 for maximum equality and 1 for maximum inequality [23]. Furthermore, for a parametric family of probability distributions, the Gini index is considered a measure of dispersion linked to the variance (Ξ_2_) [24]. Similarly, the Theil index for major parametric income distributions is expressed in terms of variance, indicating a distribution-dependent relationship between these two quantities [25]. Thus, the Theil index, often associated with variance [26], is seen as a measure of dispersion. Consequently, considering the empirical relationship between the variance Ξ_2_ and the mean (*M*_1_), that is called temporal fluctuation scaling, an expected relationship between *T* and *M*_1_ emerges, as established in the literature and termed temporal Theil scaling [27]. In fact, it has also been shown that the temporal fluctuation scaling is not always satisfied in the diffusive trajectory algorithm [27]. Therefore, in complement to the previous ideas, the inquiry persists regarding the possibility of establishing a relationship between the Hurst exponent and the temporal Theil scaling exponent within the context of the diffusive trajectory algorithm.

Regarding the temporal Theil scaling, it is important to mention that this temporal scaling is described in terms of the Shannon index and the diffusive trajectory algorithm [27]. Thus, remembering that the Shannon index is described in terms of the Theil index [13], and this in turn in terms of the generalized entropy index *GE*(*q*) where *q* is associated to *q*-th moment of the probability distribution of income of a population [20], the question naturally arises as to how to extend the Shannon index so that it depends on the *q*-th moment of the probability distribution since this opens the possibility of exploring new types of temporal scaling. Nevertheless, this must be done carefully, keeping in mind that the generalized entropy index has a removable discontinuity for *q* = 0 and *q* = 1. Also, it is observed that in principle the only requirement to calculate this Shannon index is that the probability distribution has a support of positive real values as it happens for a positive definite measure as a probability measure.

Multifractality is a concept that extends traditional ideas of fractality locally within a system F [28], originated as a neologism coined by Frish and Parisi in their work on turbulence in 1983 [29, 30]. This term was further affirmed by Mandelbrot [30]. Over time, multifractality has been explored through two prominent approaches: the Structure Functions Approach (SFA) and the Partition Function Approach (PFA). In the case of the SFA, this is proposed by Kantelhardt et al. in 2002 [31], defining the structure functions in terms of the *q*-th moment of the probability distribution associated with time-lagged increments *δ* in the system F. Then, a power law relationship is then assumed for these structure functions to link the exponents to the generalized Hurst exponent *H*(*q*), generalized fractal dimension *D*_*q*_, or the mass exponent function *τ*(*q*) [31, 32].

In contrast, the PFA, introduced by Grassberger and Procaccia in 1983 [33], defines a partition function in terms of a probability measure $f_{F}$ within the system F, akin to the usual ensembles of statistical mechanics. The behavior of the system is characterized by calculating the generalized fractal dimension in terms of the Renyi entropy [33]. In fact, *q* = 0, *q* = 1, and *q* = 2 correspond to the usual fractal dimension [34], the dimension of information calculated with Shannon’s entropy [35], and the dimension of correlation [33], respectively.

Furthermore, several studies in the literature have concentrated on examining and elucidating certain properties regarding the potential relationship between entropy measures and long-range memory measures, either implicitly through the auto-correlation function [36–39] or explicitly via the Hurst exponent [40]. Notably, the probability of a particular pattern *p*_*π*_(*d*), characterized by a delay *d* and a permutation *π*, being reiterated within a time series enables the establishment of properties akin to the temporal correlation of the series in a much-simplified way [36]. For instance, analytical expressions are derived for fractional Brownian motion and processes with stationary Gaussian increments, where the auto-correlation function hinges solely upon the probabilities of patterns of order *p*_*π*_(*d* = 2) [36, 37]. Nevertheless, pattern order analysis encounters complexities, particularly when the pattern’s order surpasses four, leading to intricate or dependent on many parameters at the computational level functions [36, 37].

Moreover, the generalized fractal dimension of a system serves as a direct measure of the system’s entropy [38, 40], suggesting an inherent connection between Renyi entropy and the generalized fractal dimension [33, 39, 40]. The above has motivated the interest in employing entropic analyses, such as multi-scale entropy analysis, to establish a direct linkage between the Hurst exponent of fractional Brownian motion and the sample entropy (*S*_*E*_), assuming that *S*_*E*_ conforms to a *q*-exponential function [40]. Yet, to obtain a broader perspective of the relationship between entropy as an indirect measure of a system’s fractality, it is imperative to recognize several limitations in the existing literature.

Firstly, the most common entropy measures, like Renyi entropy, are non-extensive quantities in the physical realm. This non-extensivity implies that the total entropy of a system is not computed as the sum of individual entropies when the system is divided into several regions. Consequently, entropy is not a scale-invariant or self-similar measure, which is fundamental in a fractal system. Secondly, while the diffusive trajectory algorithm measures the Hurst exponent of a fractional Brownian motion exactly [7], it prompts questions regarding the feasibility of extending such an analogy to systems that do not satisfy a fractional Brownian motion. Thus, the possibilities of extending these approaches to normalized measures, such as the Shannon index, are raised. Also, it is highlighted that this extension would arise naturally with the temporal Theil scaling exponent as the latter is linked to measures such as the Shannon index and the diffusive trajectory algorithm [27]. Accordingly, to establish a clearer relationship of entropy as a measure of the fractality of a system, one solution is to establish a connection between the generalized Hurst exponent and the temporal Theil scaling exponent as addressed in this work.

Therefore, in this paper, a review of the theoretical approaches used to address multifractality is made. From the above, an extension of the Shannon index is proposed, and then a novel theoretical relationship is deduced between the generalized Hurst exponent with a newly introduced multifractal exponent termed the *multifractal exponent of the generalized Shannon index*. To do this, the concept of multifractality within a system F is defined in Section 2, delving into two schemes for computing multifractal exponents: the structure function approach and the partition function approach. Actually, one notable multifractal exponent is the generalized Hurst exponent *H*(*q*), where *q* represents the *q*-th moment of the associated probability distribution with a measurement on the system F. Later, in section 4 the extension of the Shannon index taking into account the generalized entropy index is made. Thus, this extension is given the name *generalized Shannon index* and allows to define a newly multifractal exponent *β*_*TTS*_(*q*) in such a way that the generalized Hurst exponent is related to the temporal Theil scaling exponent as shown in Eq (31) from the subsection 5. In Section 6, the proposed relation is validated for fractional Brownian motion, and Section 7 demonstrates its application to a two financial time series, offering a optimal selection algorithm for the most significant *q*-th moment in estimating the generalized Hurst exponent.

## 2 Multifractality in complex systems

Fractality typically pertains to the geometric attributes of an object, emphasizing properties like self-similarity, scale invariance, and nowhere differentiable, with its fractal dimension *D* surpassing the topological dimension [5, 41]. In other words, a fractal object exhibits recurring patterns across scales, featuring irregularities that lack smoothness in terms of differential calculus. Also, its length, area, or volume scales following a power law, where the exponent is a non-integer value surpassing the conventional dimension [5, 41]. Indeed, the characterization of the *fractal dimension D* typically employs the *box counting* method. This approach involves counting the units necessary to cover the fractal set denoted by *N*, considering a unit size *δ* > 0 as the measuring scale [34]. Thus, assuming [34]

$$\begin{array}{c} N∼\delta^{-D}, \end{array}$$ (1)

it follows that the fractal dimension is the slope of a log-log graph of the number of units needed to cover F as a function of the unit of measure *δ*. It is important to mention that there are other measures to describe fractality and that they have emerged in different branches of science such as the information dimension [35], the correlation dimension [33], the Lyapunov dimension [42], the Higuchi dimension [43], or the Haussdorf dimension [44].

Although, there are fractal systems where a single fractal dimension is not enough to describe the collective behavior of the whole system and in such a case the concept of multifractality is reached. Specifically, a system F is considered multifractal if given a measure $f_{F}$ on the system it is satisfied that

$$\begin{array}{c} f_{F}\left(x+\delta \right)-f_{F}\left(x\right)∼\delta^{y(x)}, \end{array}$$ (2)

where *x* is a specific instant of time *t* or spatial point $\vec{r}$ under observation, ||*δ*|| > 0, and *y*(*x*) is called the singularity exponent [28].

Thence, multifractality is a property wherein a system exhibits local fractal behavior based on the specific instant of time *t* or spatial point $\vec{r}$ under observation [28]. Historically, the term multifractality was first coined by Frish and Parisi in their 1983 work on turbulence [29, 30], a concept later confirmed by Mandelbrot [30]. Concurrently, in the same year, Grassberger and Procaccia introduced the concept, utilizing Renyi entropy [33]. Presently, two predominant approaches, known as the *partition function approach (PFA)* and the *structure functions approach (SFA)*, are employed to explore and understand multifractality.

In the SFA, proposed by Kantelhardt et. al in 2002 [31], it is found that the increments of the measure $f_{F}$ characterize the entire behavior of the system, i.e, estimating the *q*-th moment of the probability distribution associated with the measure $f_{F}$ is satisfied for all *δ* > 0 that

$$\begin{array}{c} K\left(q,\delta \right)=\frac{E[|f_{F}\left(t+\delta \right)-f_{F}\left(t\right){|}^{q}]}{E[|f_{F}\left(t\right){|}^{q}]}∼\delta^{qH(q)}, \end{array}$$ (3)

where *H*(*q*) is the generalized Hurst exponent [31, 32]. Furthermore, when *H*(*q*) is constant, the system is said to have monofractal behavior and *H*(*q*) = *H* corresponds to the usual Hurst exponent, whereas if *qH*(*q*) is a nonlinear function then the system has multifractal behavior and is a strong argument against the Brownian, Fractional Brownian, Lévy and Fractional Lévy models, which are additive models, so *qH*(*q*) are portions of straight lines [32]. It goes without saying that it is also a known fact that a possible explanation for the origin of multifractality is using geometric Tweedie models [45, 46].

On the other hand, in the PFA using the same idea of box counting in fractality, it is assumed that the measure $f_{F}$ is a probability measure such that if the system F is divided into boxes of size *δ* > 0 the following two equalities are satisfied

$$f_{F}\left(\delta ,s\right)=\int_{\sigma \in B(\delta ,s)}\frac{df_{F}\left(\sigma \right)}{d\sigma }d\sigma ,$$ (4)

$$\sum_{s}f_{F}\left(\delta ,s\right)=\int_{F}\frac{df_{F}\left(\sigma \right)}{d\sigma }d\sigma =1,$$ (5)

where B(δ,s) is the *s*-th box of *δ* > 0 size in the system. Thus, the *partition function* $\Omega_{F}\left(q,\delta \right)$ is defined by [28, 33]

$$\begin{array}{c} \Omega_{F}\left(q,\delta \right)=\sum_{s}[f_{F}\left(\delta ,s\right){]}^{q}. \end{array}$$ (6)

Thence, if the Renyi entropy I(q,δ) is defined as [33]

$$\begin{array}{c} I\left(q,\delta \right)=\underset{\lambda \to q}{\text{lim}}\;\frac{1}{1-\lambda }\;\text{ln}\;(\Omega_{F}\left(\lambda ,\delta \right)), \end{array}$$ (7)

and the *generalized fractal dimension D*_*q*_ is [28, 33]

$$\begin{array}{c} D_{q}=\underset{\delta \to 0}{\text{lim}}\;\frac{I(q,\delta )}{\text{ln}\;(\delta^{-1})}=\{\begin{array}{cc} {\text{lim}}_{\delta \to 0}\;\frac{1}{q-1}\;\frac{\text{ln}\;(\Omega_{F}\left(\lambda ,\delta \right))}{\text{ln}\;\delta } & ,\;\text{if}\;q\neq 1\\ {\text{lim}}_{\delta \to 0}\;\frac{\sum_{s}f_{F}\left(\delta ,s\right)\;\text{ln}\;f_{F}\left(\delta ,s\right)}{\text{ln}\;\delta } & ,\;\text{if}\;q=1 \end{array}. \end{array}$$ (8)

An important fact to note here is that the generalized fractal dimension with *q* = 0, *q* = 1, and *q* = 2 corresponds to the usual fractal dimension [34], the information dimension calculated with Shannon entropy [35], and the correlation dimension [33], respectively. Therefore, from the generalized fractal dimension *D*_*q*_, the generalized Hurst exponent *H*(*q*) is defined as [28, 33]

$$\begin{array}{c} H\left(q\right)=\underset{\lambda \to q}{\text{lim}}\frac{\lambda D_{\lambda }-D_{\lambda }+1}{\lambda }. \end{array}$$ (9)

Finally, to complete the whole panorama of multifractality, it is crucial to highlight two additional functions that gauge the degree of multifractality within the system. These are referred to as the *mass exponent function τ*(*q*) and the singularity spectrum *ϑ*(*y*). The mass exponent function is related with the generalized Hurst exponent as follows [28, 33]

$$\begin{array}{c} \tau (q)=qH(q)-1. \end{array}$$ (10)

In the same way, the singularity spectrum is expressed in terms of the mass exponent function through the Legendre transform, that is,

$$\begin{array}{c} \tau \left(q\right)=yq-\vartheta \left(y\right)=q\frac{d\tau }{dq}-\vartheta \left(y\left(q\right)\right). \end{array}$$ (11)

Indeed, the singularity spectrum *ϑ*(*y*) corresponds to a concave function that gathers all the singularity exponents or singularity strengths *y*(*t*) of the system [28, 33].

## 3 Multifractal Detrended Fluctuation Analysis

Now, to grasp the computation of the generalized Hurst exponent in empirical time series data, the *Multifractal Detrended Fluctuation Analysis* (MF-DFA) method is presented [28, 31, 32]. It is essential to note that various methods exist for calculating the generalized Hurst exponent, including the detrended moving average (DMA) [47], geometric method-based procedures (GM algorithms) [9], absolute value method (AVE) [10], fractal dimension algorithms (FD) [48], Generalized Hurst Exponent (GHE) [49], triangle total areas (TTA) [12], and the KS method [50]. Nevertheless, preference is given over the MF-DFA considering that for this generalized Hurst exponent method its canonical measure function is known [51].

Multifractal Detrended Fluctuation Analysis (MF-DFA) is a method designed on innovation time series such that the generalized Hurst exponent *H*(*q*) is computed over a time series $\{Z(t){\}}_{t=1}^{N}$ with the following steps [31]:

1. Determine the profile of the time series as
  $$\begin{array}{c} U\left(t\right)=\sum_{k=1}^{t}[Z\left(k\right)-E[Z(t)]]. \end{array}$$ (12)
2. Divide the profile *U*(*t*) into *N*_*δ*_ = ⌊*N*/*δ*⌋ non-overlapping segments of length *δ* > 0. Thus, if the time series is not a multiple of the considered time scale *δ* > 0, then the same procedure is repeated over time series starting from the opposite end.
3. Calculate the local trend $\tilde{U}$ for each of the *N*_*δ*_ segments (2*N*_*δ*_ if N/δ∉N) using some approximation technique, such as a detrended fluctuation analysis (DFA or polynomial fitting), or a detrended moving average (DMA). The detrended residuals $\{\epsilon (t){\}}_{t=1}^{N}$ are defined by
  $$\begin{array}{c} \epsilon \left(t\right)=U\left(t\right)-\tilde{U}\left(t\right). \end{array}$$ (13)
4. Estimate the variance for each of the *N*_*δ*_ segments (2*N*_*δ*_ if N/δ∉N) as
  $$\begin{array}{c} F^{2}\left(\delta ,s\right)=\{\begin{array}{cc} \delta^{-1}\sum_{k=1}^{\delta }[\epsilon ((s-1)\delta +k){]}^{2} & ,\;\text{if}\;1\leq s\leq N_{\delta }\\ \delta^{-1}\sum_{k=1}^{\delta }[\epsilon (N-(s-N_{\delta })\delta +k){]}^{2} & ,\;\text{if}\;N_{\delta }<s\leq 2N_{\delta } \end{array}, \end{array}$$ (14)
  for the *s*-th segment of size *δ* > 0. It is important to note that in the second line of Eq (14), there is the restriction *s* > *N*_*δ*_.
5. Average over all segments to obtain the *q*-th order overall detrended fluctuation function
  $$\begin{array}{c} F\left(q,\delta \right)=\{\begin{array}{cc} {}[\frac{1}{2N_{\delta }}\sum_{s=1}^{2N_{\delta }}[F^{2}\left(\delta ,s\right){]}^{q/2}{]}^{1/q} & ,\;\text{if}\;q\neq 0\\ \text{exp}(\frac{1}{2N_{\delta }}\sum_{s=1}^{2N_{\delta }}\text{ln}[F(\delta ,s)]) & ,\;\text{if}\;q=0 \end{array}=\underset{q\to \lambda }{\text{lim}}[\frac{1}{2N_{\delta }}\sum_{s=1}^{2N_{\delta }}F^{\lambda }\left(\delta ,s\right){]}^{1/\lambda }. \end{array}$$ (15)
6. Repeat steps 2 to 5 for several time scales *δ*.
7. Determine the scaling behavior of the *q*-th order overall detrended fluctuation by analyzing log-log plots F(q,δ) versus *δ* > 0 for each value of q∈R. If the time series $\{Z(t){\}}_{t=1}^{N}$ are long-range power-law correlated, F(q,δ) increases, for large values of *δ* > 0, as a power-law [31],
  $$\begin{array}{c} F\left(q,\delta \right)∼\delta^{H(q)}, \end{array}$$ (16)
  where *H*(*q*) is the generalized Hurst exponent.

Here it is worth mentioning that for values *q* > 0, the behavior of the generalized Hurst exponent is dominated by the segments with the highest variance *F*^2^(*δ*, *s*) (see Eq (14)), which implies that *H*(*q*) describes the scaling behavior of segments with large fluctuations. In the same way, for values *q* < 0, the generalized Hurst exponent *H*(*q*) explains the scaling behavior of segments with small fluctuations, which are usually characterized by a higher exponent [28, 31]. Now, if the form of the expressions (6) and (15) is compared, a high similarity is observed to the point that it is affirmed that the partition function of the MF-DFA method is

$$\begin{array}{c} \Omega_{MF}\left(q,\delta \right)\equiv \Omega_{F}^{\left(MF-DFA\right)}\left(q,\delta \right)=\sum_{s=1}^{N_{\delta }}F^{q}\left(\delta ,s\right). \end{array}$$ (17)

Indeed, defining a canonical measure *μ*(*q*, *δ*, *s*) = *F*^*q*^(*δ*, *s*)/Ω_*MF*_(*q*, *δ*) in terms of this partition function and a simple moving average, the generalized fractal dimension *D*_*q*_ (see Eq (8)), the singularity strength $y\left(q\right)=\frac{d\tau }{dq}$, and the singularity spectrum *ϑ*(*y*) (see Eq (11)) are calculated [51]. Also, it is worth noting that many times the fluctuations or variances that are observed locally in the residuals of the system, measured through *F*^2^(*δ*, *s*), do not exceed the value of 1, which implies that the partition function (17) satisfies the expressions (4) and (5) as required for the PFA.

Finally, this section is concluded by presenting a stochastic process that is defined with the classical Hurst exponent called *fractional brownian motion (fBm)*. Fractional Brownian motion is the generalization of Brownian motion and first introduced in 1953 by P. Lévy although the name was actually given by B. Mandelbrot once he recognized its importance [52, 53]. Actually, the fractional Brownian motion with Hurst exponent *H* = *H*(*q* = 2), denoted by {*B*_*H*_(*t*)}_*t*≥0_, is characterized by the following four properties [52]:

1. It has zero mean at all times, that is, $E[B_{H}\left(t\right)]=0$, for all *t* ≥ 0.
2. It has stationary increments, that is, the probability distribution of the increments of the distribution only depends on the elapsed time interval, independent of the time instant in which the distribution is evaluated.
3. It is self-similar with index *H*, that is, *B*_*H*_(*at*) and *a*^*H*^*B*_*H*_(*t*) have the same probability distribution for all *a* > 0.
4. It has a Gaussian distribution for its stationary increments, that is, $B_{H}\left(t\right)-B_{H}\left(s\right)∼N(0,\frac{{\left(t-s\right)}^{H}}{\sqrt{2H}\Gamma \left(H+1/2\right)})$, for all *t* ≥ *s* ≥ 0, where N(μ,σ) represents a normal distribution with mean *μ* and standard deviation *σ*.

It is noteworthy that, in a historical context, fractional Brownian motion was utilized even before P. Lévy’s contributions. A. N. Kolmogorov employed fractional Brownian motion in 1940 to investigate spiral curves in Hilbert space [54]. Furthermore, its applications extend to the realm of the random Fourier transform [55], and the exploration of correlations in stochastic processes with stationary increments of order *n* [56]. The significance of fractional Brownian motion grew over time, culminating in its formal definition by B. Mandelbrot and J. Van Ness in 1968, encapsulated in the stochastic fractional equation [52, 53]

BH(t)=BH(0)+I0tH+12(ξ(t))=BH(0)+1Γ(H+12)∫0t(t-τ)H-12ξ(τ)dτ, (18)

where *ξ*(*t*) is a white noise and Itatbν is the right-handed-sided Riemann-Liouville fractional integral of order $H+\frac{1}{2}>0$. Thus, if *H* = 1/2, the fractional Brownian motion is an usual Brownian motion, if *H* > 1/2, the increments of the process are positively correlated, and if *H* < 1/2, the increments of the process are negatively correlated. Here it is worth mentioning that the Hurst index is a measure of long-range memory in time series. Thus, it is related to the auto-correlation of the time series and the rate at which it decreases as the delay between pairs of values increases.

## 4 Generalized Shannon index

In the preceding section, multifractality was identified as an expansion of the fractal characteristics of a system F, wherein the fractal behavior is contingent upon the observation point, specifically on the neighborhood in which it evaluates the singularity exponent *y*(*t*). Thus, various multifractality concepts involve the definition of limits and, consequently, of neighborhoods around a point (refer to Eqs (7), (8) and (9)). Thence, to delve into the theoretical understanding of the relationship with the temporal Theil scaling exponent *α*_*TTS*_(*t*), it becomes imperative to extend the Shannon index through the utilization of the generalized entropy index *GE*(λ). This extension broadens the Theil index’s definition and the foundations of temporal Theil scaling. Now, we define the generalized entropy index *GE*(λ) as [20]

$$\begin{array}{c} GE\left(\lambda \right)=\{\begin{array}{cc} -\frac{1}{N}\sum_{i=1}^{N}\text{ln}(\frac{y_{i}}{\bar{y}}) & ,\;\text{if}\;\lambda =0\\ \frac{1}{N}\sum_{i=1}^{N}\frac{y_{i}}{\bar{y}}\text{ln}(\frac{y_{i}}{\bar{y}}) & ,\;\text{if}\;\lambda =1\\ \frac{1}{N\lambda (\lambda -1)}\sum_{i=1}^{N}[{\left(\frac{y_{i}}{\bar{y}}\right)}^{\lambda }-1] & ,\;\text{if}\;\lambda \notin \{0,1\} \end{array}=\underset{q\to \lambda }{\text{lim}}\frac{1}{q(q-1)}[\frac{E[y^{q}]}{E^{q}\left[y\right]}-1], \end{array}$$ (19)

where *N* represents the size of the population, *y*_*i*_ is the income for case *i*, and $\bar{y}$ is the sample average.

Hence, with small values of λ, the generalized entropy index *GE*(λ) exhibits sensitivity to minor incomes, while larger λ values render the index sensitive to major incomes. Notably, when λ = 1, the generalized entropy index *GE*(λ) corresponds to the Theil index *T*. Also, the Shannon index T was defined as a normalization of the Theil index [57]. Therefore, if it is observed that this normalization value is made with respect to the maximum value that the Theil index can take, then the *generalized Shannon index* S(λ) is defined as

$$\begin{array}{cc} S(\lambda ) & =GE_{max}\left(\lambda \right)-GE\left(\lambda \right)=\underset{q\to \lambda }{\text{lim}}\frac{N^{q-1}}{q(q-1)}-1-GE\left(\lambda \right)\\ & =\underset{q\to \lambda }{\text{lim}}\frac{1}{q(q-1)}[\frac{N^{q}}{N}-1-(\frac{E[y^{q}]}{E^{q}\left[y\right]}-1)]\\ & =\underset{q\to \lambda }{\text{lim}}\frac{N^{q-1}}{q(q-1)}[1-\sum_{k=1}^{N}{\left(\frac{y_{k}}{\sum_{j=1}^{N}y_{j}}\right)}^{q}]. \end{array}$$ (20)

Thus, changing the dummy variable λ to *q* and defining the normalized income $x_{k}=y_{k}/\sum_{j=1}^{N}y_{j}$, it is clear that the generalized Shannon index is

$$\begin{array}{c} S\left(q\right)=\underset{\lambda \to q}{\text{lim}}\frac{N^{\lambda -1}}{\lambda (\lambda -1)}[1-\sum_{k=1}^{N}x_{k}^{\lambda }]. \end{array}$$ (21)

Note that Eq (21), due to the way it was defined, is maximum when there is maximum equality in the income distribution, that is, *x*_*k*_ = 1/*N*, for all *k* ∈ {1, 2, …, *N*}. From this, taking a time series of positive values ${\left\{Z_{t}\right\}}_{t=1}^{N}$, the diffusive trajectory algorithm generates multiple time sub-series *X*(*t*, *s*), such that the generalized Shannon index at time *t* is

$$\begin{array}{c} S\left(q,t\right)=\underset{\lambda \to q}{\text{lim}}\frac{{\left(N-t+1\right)}^{\lambda -1}}{\lambda (\lambda -1)}[1-\sum_{s=0}^{N-t}[X(t,s){]}^{\lambda }], \end{array}$$ (22)

where X(t,s) is the normalized *s*-th diffusive trajectory defined as

$$\begin{array}{c} X\left(t,s\right)=\frac{X(t,s)}{\sum_{s=0}^{N-t}X\left(t,s\right)}=\frac{\sum_{i=1}^{t}Z_{i+s}}{\sum_{s=0}^{N-t}\sum_{i=1}^{t}Z_{i+s}},\;\text{with}\;s\in \{0,1,\ldots ,N-t\}. \end{array}$$ (23)

In Eq (23), $X\left(t,s\right)=\sum_{i=1}^{t}Z_{i+s}$ is known as the *s*-th diffusive trajectory of ${\left\{Z_{t}\right\}}_{t=1}^{N}$. Finally, the temporal Theil scaling is defined as the following power law between the normalized Shannon index and the normalized mean of each diffusive trajectory [27]

$$\begin{array}{c} \frac{S(1,t)}{S_{max}\left(1\right)}=K_{TTS}\left(t\right){\left|1-\frac{M_{1}\left(t\right)}{M_{1,max}}\right|}^{\alpha_{TTS}\left(t\right)} \end{array}$$ (24)

where *M*_1_(*t*), *K*_*TTS*_(*t*) and *α*_*TTS*_(*t*) are the mean of the *s*-th diffusive trajectory, the constant and exponent of the temporal Theil scaling, respectively. Additionally,

$$\begin{array}{c} S_{max}\left(q\right)=\text{max}\{S(q,t):\;1\leq t\leq N\}, \end{array}$$ (25)

$$\begin{array}{c} M_{1,max}=\text{max}\{M_{1}\left(t\right):\;1\leq t\leq N\}. \end{array}$$ (26)

## 5 Temporal Theil scaling exponent as multifractal exponent

Now, with the extension made to the Shannon index T, notice that Eq (21) is similar to Renyi entropy (see Eq (7)) in the sense that both are measures of entropy over an arbitrary complex system or data set as a time series. Besides, the Renyi entropy is not an extensive quantity in the physical sense, which implies that paradoxes such as the Gibbs paradox are generated [58], while the generalized Shannon index is a homogeneous function of degree *q*, that is, if all the values in the system increase by an amount *β* > 0 it follows that

$$\begin{array}{c} S\left(q;\beta y_{k}\right)=\underset{\lambda \to q}{\text{lim}}\frac{{\left(\beta N\right)}^{\lambda -1}}{\lambda (\lambda -1)}[1-\sum_{k=1}^{N}x_{k}^{\lambda }]=\underset{\lambda \to q}{\text{lim}}\;\beta^{\lambda -1}S\left(\lambda \right)∼\beta^{q-1}S\left(q\right), \end{array}$$

since the maximum value is now proportional to *β*^*q*−1^*N*^*q*−1^, while the normalized income does not change under scaling. Thus, the generalized Shannon index is a more precise quantity to use in describing the scale invariance of a system as well as not having the Gibbs paradox problem when *q* = 1. Therefore, it is useful to be able to extend the ideas of multifractality by means of the generalized Shannon index in such a way that if the expressions (6), (7), (17) and (22) are compared, the partition function of the generalized Shannon index is defined by analogy as

$$\begin{array}{c} \Omega_{TTS}\left(q,\delta \right)=1-\sum_{s=0}^{N-\delta }[X(\delta ,s){]}^{q}=1-\sum_{s=0}^{N-\delta }[\frac{X(\delta ,s)}{\sum_{k=0}^{N-\delta }X\left(\delta ,k\right)}{]}^{q}. \end{array}$$ (27)

It is important to note that the partition function (27) has a functional form already similar to that of the temporal Theil scaling. Although, a last step is missing regarding normalizing the generalized Shannon index with respect to the maximum value in time, that is, $S_{max}\left(q\right)$.

Returning to the Eqs (22) and (27), it is clear that any type of normalization with respect to the maximum in the partition function of the generalized Shannon index depends on the size of the system, that is, on the length of the time series, and in this way it is assumed in forward that

$$\begin{array}{cc} \frac{S(q,\delta )}{S_{max}} & =\underset{\lambda \to q}{\text{lim}}[(\frac{N-\delta +1}{N-\delta_{max}+1}{)}^{\lambda -1}\frac{\Omega_{TTS}\left(\lambda ,\delta \right)}{\Omega_{TTS}\left(\lambda ,\delta_{max}\right)}]\\ & =(\frac{N-\delta +1}{N-\delta_{max}+1}{)}^{q-1}\frac{1-\sum_{s=0}^{N-\delta }X^{q}\left(\delta ,s\right)}{1-\sum_{s=0}^{N-\delta }X^{q}\left(\delta_{max},s\right)}\\ & ∼[1-\frac{M_{q}\left(\delta \right)}{M_{q,max}}{]}^{\lambda_{TTS-MF}\;q[H(q)+1]-\kappa_{TTS-MF}\;D_{0}}. \end{array}$$ (28)

where *δ*_*max*_ denotes the time step where generalized Shannon index is maximum, *M*_*q*_(*δ*) is the *q*-th moment of the diffusive trajectories time series at time *δ*, *M*_*q*,*max*_ = max_*δ*_{*M*_*q*_(*δ*)}, *D*_0_ is the fractal dimension of the system or usual dimension, λ_*TTS*−*MF*_ > 0 is the coupling parameter of the generalized Hurst exponent with the temporal Theil scaling exponent, and $\kappa_{TTS-MF}\in R$ is the coupling parameter of the usual dimension with the temporal Theil scaling exponent. Also, note that these coupling parameters arise as an effect of the size of the system, that is, the length of the time series *N*.

Therefore, it is now possible to define a new multifractal exponent based on generalized Shannon index partition function and in such a way that the temporal Theil scaling exponent corresponds to a particular case of it. Thus, the multifractal exponent of the generalized Shannon index is defined as

$$\begin{array}{c} \beta_{TTS}\left(q\right)=\underset{\delta \to \delta_{max}}{\text{lim}}\frac{\text{ln}(\frac{S(q,\delta )}{S(q,\delta_{max})})}{\text{ln}\;\theta_{c}\left(q,\delta \right)}=\lambda_{TTS-MF}\;q[H(q)+1]-\kappa_{TTS-MF}\;D_{0}, \end{array}$$ (29)

where

$$\begin{array}{c} \theta_{c}\left(q,\delta \right)=1-\frac{M_{q}\left(\delta \right)}{M_{q,max}}=1-\frac{M_{q}\left(\delta \right)}{M_{q}\left(\delta_{max}\right)}, \end{array}$$ (30)

is the critical parameter associated with an ordered phase in a condensed matter phase transition [27, 59, 60]. Observe that the limit of Eq (29) is with respect to time *δ* → *δ*_*max*_ which is well defined since the time instant in which the maximum occurs for the generalized Shannon index is the same for the moments around the origin of the distribution. In fact, said instant of time, as seen at the beginning of this section, corresponds to the moment in which the diffusive trajectory has maximum equality in analogy to an income distribution, which implies the lowest generalized entropy index over time for the multiple diffusive paths.

Finally, if Eq (29) is applied for *q* = 1, the temporal Theil scaling exponent in terms of the generalized Hurst exponent *H*(*q*) is

$$\begin{array}{cc} \alpha_{TTS} & \equiv \underset{q\to 1}{\text{lim}}\;\beta_{TTS}\left(q\right)=\underset{q\to 1}{\text{lim}}[\lambda_{TTS-MF}\;q[H(q)+1]-\kappa_{TTS-MF}\;D_{0}]\\ & =\lambda_{TTS-MF}(H(q=1)+1)-\kappa_{TTS-MF}D_{0}\\ & =\lambda_{TTS-MF}(H+1)-\kappa_{TTS-MF}D_{0}, \end{array}$$ (31)

where the last step is only valid in monofractal systems such as the fractional Brownian movement with Hurst exponent *H*.

## 6 Temporal Theil scaling in fractional Brownian motion

This section verifies the validity of the generalized Shannon index multifractality approach proposed in the previous section. To do this, starting from Eq (31), the simulation of multiple fractional Brownian motion trajectories is performed and its exponent of the temporal Theil scaling is calculated to corroborate that the found expression works in this type of stochastic process. With the above, the process to follow to corroborate Eq (31) is:

1. Simulate *N*_*fBm*_ fractional Brownian motion trajectories by setting *T*_*fBm*_ time steps for different values of the Hurst exponent *H*. For this, the following aspects are taken into account:
  - The distribution of increments of a fractional Brownian motion, i.e. *g*_*H*_(*t*) = *B*_*H*_(*t* + 1) − *B*_*H*_(*t*), is called *fractional Gaussian noise*, and the latter has already been computationally implemented quite efficiently in Python [61]. Indeed, the implementation process outlined in Python by Rydin Gorjao et al. follows a rough procedure [61]. Initially, a circulant covariance matrix is defined, with its entries determined by the covariance function given by $\gamma \left(k\right)=\frac{1}{2}[|k-1{|}^{2H}+|k+1{|}^{2H}-2|k{|}^{2H}]$, for all *k* ≥ 0. Subsequently, this matrix is diagonalized. Finally, two identically distributed standard normal random variables are generated, and the fast Fourier transform is applied in conjunction with the eigenvalues and eigenvectors of the circulant covariance matrix [62]. This process yields a sample of fractional Brownian motion by utilizing the accumulated increments of the fractional Gaussian noise sample mentioned earlier. Notably, this technique is recognized in the literature as the Wood-Chan or Davies-Harte method [62–64].
  - The estimate of the Hurst exponent in short time series has a high uncertainty as it has already been shown in [65]. Then, a selection criterion is generated on each fractional Brownian motion path such that if the computationally found Hurst exponent, say *H*_*exp*_, is in the range [*H* − *ε*_*fBm*_, *H* + *ε*_*fBm*_], then the simulation is a valid simulation. Otherwise, the process is repeated until completing the *N*_*fBm*_ simulations. Here it is worth mentioning that once the sample of a fractional Brownian motion with Hurst exponent *H* is generated in the previous step, the computational Hurst exponent *H*_*exp*_ is estimated in each simulation through the MF-DFA method mentioned in the section 3 and implemented in Python by Rydin Gorjao et. al. through a function called *MFDFA* [61].
2. Estimate the increments of the fractional Brownian motion sample (fBm), the absolute value of the fractional Brownian motion, and the volatilities of the fractional Brownian motion for each simulation using the following definitions
  $$L_{t}=B_{H}\left(t+1\right)-B_{H}\left(t\right),$$ (32)
  $$A_{t}=|L_{t}|,$$ (33)
  $$V_{t}=\frac{1}{\text{max}\{A_{\tau }|1\leq \tau \leq N-1\}}\sqrt{\left|\frac{L_{t}-E[L_{t}]}{\sigma [L_{t}]}\right|},$$ (34)
  respectively. It is important to mention that in Eq (34), *σ*[⋅] represents the standard deviation of the random variable. Furthermore, the term inside the square root is usually known as *standard score* or *z-score*. Note that the expected value E[·] or the standard deviation *σ*[⋅] used in the definition of the *z-score* are statistical properties that are obtained at each time step from the usual formulas for the sample mean and the unbiased standard deviation of a sample, respectively. Thence, the total time series is tripled.
3. Apply the diffusive algorithm described by $X\left(t,s\right)=\sum_{i=1}^{t}B_{H}\left(i+s\right)$, for the original time series of fractional Brownian motion, absolute value of the fractional Brownian motion, and volatilities of the fractional Brownian motion.
4. Calculate the cumulative mean, and the Shannon index in each of the diffusive trajectory time series by adding new data for each time *t*.
5. Perform power law regression based on Eq (24) to find the temporal Theil scaling coefficient *K*_*TTS*_(*t*) and the temporal Theil scaling exponent *α*_*TTS*_(*t*). It is important to mention that in all the iterations of this process, one cost function is estimated on the adjustment of the temporal Theil scaling given by the coefficient of determination $R_{TTS}^{2}$.
6. Filter the simulations where $R_{TTS}^{2}$ exceeds a certain threshold value *r*_*fBm*_ ≥ 0 and average the values obtained for the temporal Theil scaling exponent in the different simulations of a fractional Brownian motion with Hurst exponent *H*. Thus, when repeating the process with different values of the Hurst exponent, we have the average of the temporal Theil scaling exponent *α*_*TTS*_(*H*) as a function of the Hurst exponent *H*.
7. Perform linear regression based on Eq (31) to find the coupling parameter of the generalized Hurst exponent with the temporal Theil scaling exponent λ_*TTS*−*MF*_ > 0 and the coupling parameter of the usual dimension with the temporal Theil scaling exponent $\kappa_{TTS-MF}\in R$. It is important to mention that in all the iterations of this process, two cost function is estimated on the adjustment of Eq (31) given by the coefficient of determination *R*^2^ and the *p*-th mean absolute error *MAE*_*p*_(*n*_*H*_) defined by
  $$\begin{array}{c} MAE_{p}\left(n_{H}\right)=\{\begin{array}{cc} {}[\frac{1}{n_{H}}\sum_{k=1}^{n_{H}}|e_{k}{|}^{p}{]}^{\frac{1}{p}} & ,\;\text{if}\;p\neq 0\\ \text{exp}(\frac{1}{2n_{H}}\sum_{k=1}^{n_{H}}\text{ln}|e_{k}{|}^{2}) & ,\;\text{if}\;p=0 \end{array}, \end{array}$$ (35)
  where *n*_*H*_ is the number of the different values used for the Hurst exponent, and *e*_*k*_ are the residuals obtained after adjusting for least squares.

Finally, before going on to the results after having done this process, it is worth mentioning that the parameters selected for the simulations were *N*_*fBm*_ = 200, *T*_*fBm*_ = 512, Δ*t* = 0.001, *ε*_*fBm*_ = 0.02, *r*_*fBm*_ = 0.98, *H* ∈ {0.4, 0.46, 0.52, 0.58, 0.64, 0.7, 0.76, 0.82, 0.88} (then *n*_*H*_ = 9). Regarding the values chosen for the Hurst exponent, it goes without saying that values are taken where the Hurst exponent is persistent (*H* ≳ 0.5) since the MF-DFA method for multifractality becomes imprecise for signals strongly anti-correlated when *H*(*q*) is close to zero. Also, taking into account some methods developed for the estimation of the Hurst exponent with short time series that use the entropy of Shannon, the values of *H* ∼ 0.9 are limited to avoid the problem of bias of the diffusive trajectory algorithm [66]. In addition, all the codes made are published in the Github [67]. At last, to obtain the uncertainty in the parameters estimated by the least squares regression in some adjustments, the covariance matrix *Cov*(⋅) is calculated with the vector of residuals of the fit $\vec{e}$, that is, $Cov\left(\cdot \right)\propto \vec{e}{\vec{e}}^{T}$. Then, the square root of the respective term on the diagonal of the covariance matrix is taken as the error in the fit parameters.

**Fig 1:**
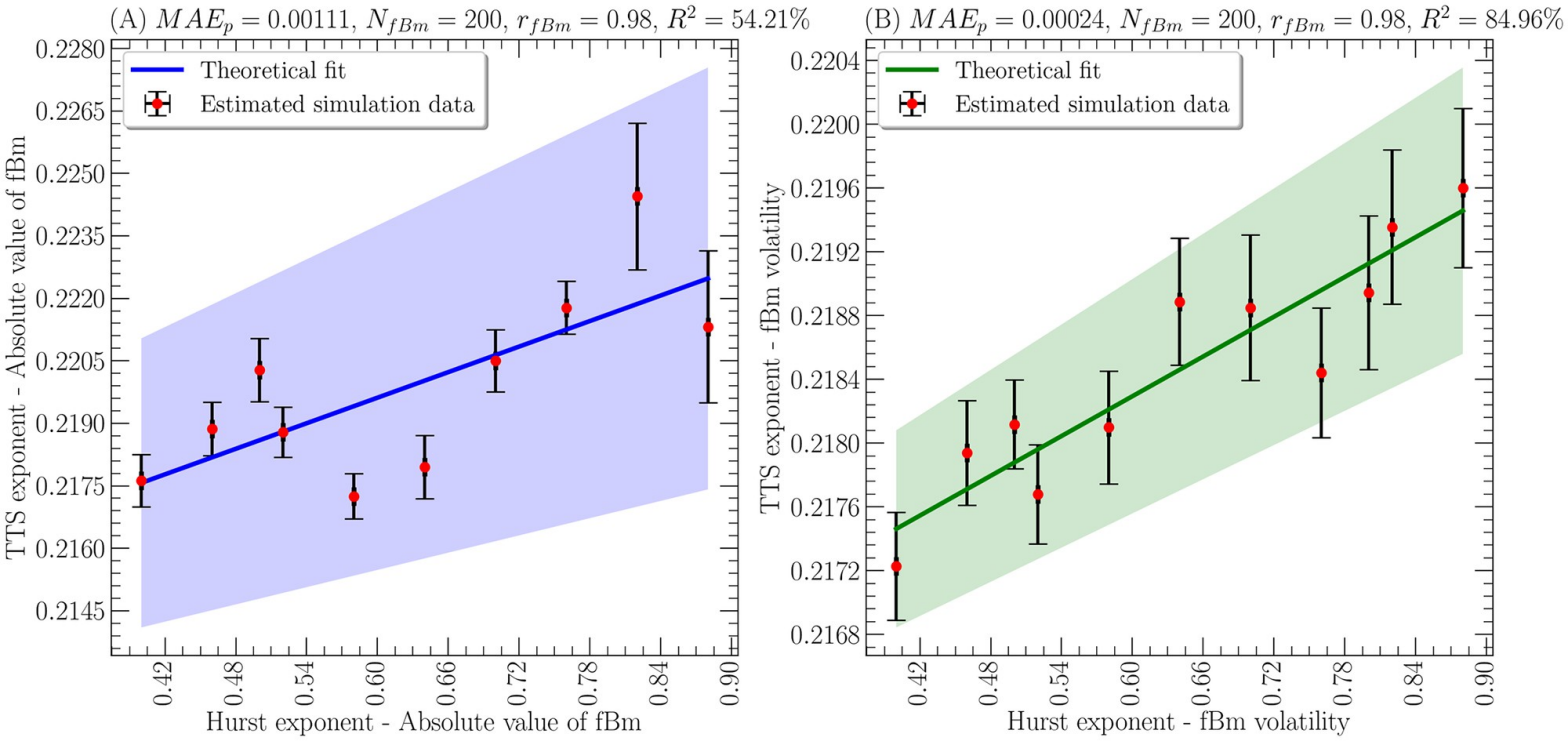
Temporal Theil scaling exponent as a function of the Hurst exponent for a fractional Brownian motion (fBm). **(A)** absolute value of fractional Brownian motion. **(B)** volatilities of the fractional Brownian motion. In all cases, the simulated data was obtained after averaging over *N*_*fBm*_ = 200 simulations of *T*_*fBm*_ = 512 time steps for each value of the Hurst exponent displayed. In addition, the error bars were taken with the standard deviations generated by the different simulations and the theoretical fitting to Eq (31) as a continuous line.

Fig 1 illustrates the temporal Theil scaling exponent as a function of the Hurst exponent under the specified parameters. Notably, some datasets display a considerable standard deviation even after averaging multiple simulations, suggesting the potential for improvement through an increased number of simulations *N*_*fBm*_ or by utilizing a fractional Brownian movement with a greater number of time steps *T*_*fBm*_. However, it is essential to acknowledge the computational challenges associated with the diffusive trajectory algorithm, whose execution time and memory usage grows proportionally to $O(T_{fBm}^{2})$.

In the regression analysis outlined in (31), the coefficient of determination *R*^2^ for the time series of absolute value of the fractional Brownian motion and the time series of volatilities of the fractional Brownian motion are reported as 54.21% and 84.96%, respectively. This data, along with the coupling parameters obtained in each case (considering *D*_0_ = 1 for time series), is presented in Table 1. In fact, it is observed that the coupling constant between the temporal Theil scaling exponent and the fractal dimension *κ*_*TTS*−*MF*_ is negative in both cases, indicating an inversely proportional relationship between these exponents. Conversely, the coupling constant between the temporal Theil scaling exponent and the generalized Hurst exponent λ_*TTS*−*MF*_ is positive but two orders of magnitude smaller, suggesting that the multifractal exponent of the generalized Shannon index is more sensitive to changes in the fractal dimension of the fractional Brownian motion. Finally, it is crucial to highlight that the temporal Theil scaling of the original time series is not performed, as a fractional Brownian motion is not positively defined by its positive or negative increments.

**Table 1:** Parameters of the relationship between the multifractal exponent of the generalized Shannon index *β*_*TTS*_(*q*) and the generalized Hurst exponent *H*(*q*) together with its coefficient of determination *R*^2^ obtained after the fit with the expression (31) for a fractional Brownian motion (fBm).

Time series	Coupling type	Coupling constant value	*MAE*_1_ (×10^−3^)	Coefficient of determination *R*^2^(%)
Absolute value	Temporal Theil scaling and Hurst exponent	0.010225 ± 0.003322	1.107	54.21
Absolute value	Temporal Theil scaling and fractal dimension	-0.203254 ± 0.005463	1.107	54.21
fBm volatility	Temporal Theil scaling and Hurst exponent	0.004158 ± 0.000583	0.241	84.96
fBm volatility	Temporal Theil scaling and fractal dimension	-0.211641 ± 0.000968	0.241	84.96

Therefore, the multifractal exponent of the generalized Shannon index demonstrates a high degree of precision for a fractional Brownian movement, as evidenced by the satisfaction of the expression (31), especially in the case of volatilities of the fractional Brownian motion, where the coefficient of determination is *R*^2^ = 84.96%. However, the time series of absolute value of the fractional Brownian motion may appear to deviate from this trend, as indicated by the *R*^2^ value of 54.21%, but in such a case it should be remembered that the small values close to zero associated with absolute value of the fractional Brownian motion magnify the impact of small fluctuations in the time series. To address this, it is recommended to compute the generalized Hurst exponent *H*(*q*) with *q* < 0, as opposed to the conventional Hurst exponent *H*(*q* = 2) = *H*. Furthermore, remembering that the volatilities of the fractional Brownian motion definition precisely mitigates the issue of small fluctuations when the z-score is calculated (see Eq (34)).

## 7 Application of the multifractal exponent of the generalized Shannon index

Finally, this section presents the association between the temporal Theil scaling exponent and the generalized Hurst exponent in empirical time series, a relationship previously substantiated in Section 4 and validated for fractional Brownian motion in Section 6. Thence, when dealing with time series of empirical data with an unknown Hurst exponent, a comprehensive exploration is required to obtain multiple temporal Theil scaling exponents and generalized Hurst exponents from the same time series. The proposed methodology involves accumulating data from the time series to calculate multiple temporal Theil exponents and generalized Hurst exponents, thereby constructing a substantial sample of potential values for these parameters. Thus, comparing *α*_*TTS*_ and *H*(*q*) at the same instant of time, it is possible to observe the relationship between these two quantities.

For this purpose, the Taylor series of the multifractal exponent of the generalized Shannon index for a fractional Brownian motion with Hurst exponent *H*_*fBm*_ around *q* = 1 is considered, that is

$$\begin{array}{c} \beta_{fBm}\left(q\right)=\beta_{fBm}\left(1\right)+\frac{d\beta_{fBm}}{dq}{|}_{q=1}\left(q-1\right)=\alpha_{TTS}+\left(q-1\right)\lambda_{TTS-MF}(H_{fBm}+1), \end{array}$$ (36)

where $\frac{d\beta_{fBm}}{dq}{|}_{q=1}$ is calculated by Eq (29), and the Taylor series is truncated to first order by the linear relationship that was verified for the fractional Brownian motion in the section 6. Now, equating (36) with (31), and solving for *q* − 1, we obtain

$$\begin{array}{c} q-1=q-\frac{\alpha_{TTS}+\kappa_{TTS-MF}D_{0}}{\lambda_{TTS-MF}(H_{fBm}+1)}∼1-\frac{\alpha_{TTS}+\kappa_{TTS-MF}D_{0}}{\lambda_{TTS-MF}(H_{fBm}+1)},\;\text{for}\;q\to 1. \end{array}$$ (37)

The next step is to note that the generalized Hurst exponent *H*(*q*) in terms of the multifractal exponent of the generalized Shannon index *β*(*q*) satisfies that

$$\begin{array}{c} H\left(q\right)=\frac{\beta \left(q\right)+\kappa_{TTS-MF}D_{0}}{\lambda_{TTS-MF}q}-1. \end{array}$$ (38)

where the expression (29) has been used such that the multifractal exponent of the generalized Shannon index *β*(*q*) is proportional to the generalized Hurst exponent *H*(*q*). Thus, the *n*-th derivative of the generalized Hurst exponent, with n∈N, is

H(n)(q)≡dnH(q)dqn=∑j=0n(nj)djdqj(β(q)+κTTS-MFD0λTTS-MF)dn-jdqn-j(q-1)=∑j=0n(nj)djdqj(β(q)+κTTS-MFD0λTTS-MF)(-1)n-j(n-j)!q-(n-j)-1=∑j=0nΓ(n+1)Γ(j+1)djdqj(β(q)+κTTS-MFD0λTTS-MF)(-1)n-jq-(n-j)-1. (39)

Then, the Taylor series of the generalized Hurst exponent around *q* = 1 is

$$\begin{array}{cc} H(q) & =H\left(1\right)+\sum_{n=1}^{\infty }\frac{H^{\left(n\right)}\left(1\right)}{\Gamma (n+1)}{\left(q-1\right)}^{n}\\ & =H\left(1\right)+\sum_{n=1}^{\infty }\sum_{j=0}^{n}\frac{{\left(-1\right)}^{n-j}}{\lambda_{TTS-MF}\Gamma \left(j+1\right)}[\beta^{\left(j\right)}\left(1\right)+\delta_{j,0}\;\kappa_{TTS-MF}D_{0}]{\left(q-1\right)}^{n}\\ & =H\left(1\right)+\sum_{n=1}^{\infty }(\frac{\alpha_{TTS}+\kappa_{TTS-MF}D_{0}}{\lambda_{TTS-MF}}+\sum_{j=1}^{n}\frac{{\left(-1\right)}^{n-j}\beta^{\left(j\right)}\left(1\right)}{\lambda_{TTS-MF}\Gamma \left(j+1\right)}){\left(q-1\right)}^{n}\\ & =H\left(1\right)+\sum_{n=1}^{\infty }\frac{\alpha_{TTS}+\kappa_{TTS-MF}D_{0}}{\lambda_{TTS-MF}}{\left(q-1\right)}^{n}+\sum_{n=1}^{\infty }\sum_{j=1}^{n}\frac{{\left(-1\right)}^{n-j}\beta^{\left(j\right)}\left(1\right)}{\lambda_{TTS-MF}\Gamma \left(j+1\right)}{\left(q-1\right)}^{n}\\ & =H\left(1\right)+\sum_{n=1}^{\infty }\frac{\alpha_{TTS}}{\lambda_{TTS-MF}}{\left(q-1\right)}^{n}+\lambda_{TTS-MF}^{-1}R[\beta (q)] \end{array}$$

$$\begin{array}{cc} & =\frac{\kappa_{TTS-MF}D_{0}}{\lambda_{TTS-MF}}-1+\frac{\alpha_{TTS}}{\lambda_{TTS-MF}}+\sum_{n=1}^{\infty }\frac{\alpha_{TTS}}{\lambda_{TTS-MF}}{\left(q-1\right)}^{n}+\frac{R[\beta (q)]}{\lambda_{TTS-MF}}, \end{array}$$ (40)

where *δ*_*j*,0_ is the Kronecker delta, *β*(1) = *α*_*TTS*_ and the expressions (38) and (39) were used. Furthermore, we define

$$\begin{array}{c} R[\beta (q)]=\sum_{n=1}^{\infty }[\kappa_{TTS-MF}D_{0}+\sum_{j=1}^{n}\frac{{\left(-1\right)}^{n-j}\beta^{\left(j\right)}\left(1\right)}{\Gamma (j+1)}]{\left(q-1\right)}^{n}. \end{array}$$ (41)

Consequently, motivated by Eq (37), it is assumed that

$$\begin{array}{c} q-1∼1-\frac{1}{{\tilde{H}}_{fBm}+1}(\frac{\alpha_{TTS}+\kappa_{TTS-MF}D_{0}}{\lambda_{TTS-MF}}), \end{array}$$ (42)

which will be called hereinafter *local approximation of fractional Brownian motion (LA-fBm)*. Thus, it is interpreted that in a small enough neighborhood of size *ε* > 0 around *q*, the generalized Hurst exponent behaves as a fractional Brownian motion with Hurst exponent ${\tilde{H}}_{fBm}=H_{fBm}\left(q,\varepsilon \right)$. In other words, the generalized Hurst exponent is approximated by chunks of linear functions with different Hurst exponents, or equivalently, by multiple fractional Brownian motions. Consequently, the LA-fBm is an approximation in which a multifractal system is locally composed of different fractional Brownian motions such that some of them contribute more than others depending on the type of system and the time or space in which it is observed. It is important to note then that the factor that is preserved independent of LA-fBm is $\alpha_{TTS}\lambda_{TTS-MF}^{-1}$, which implies that the expression (40) is written as

$$\begin{array}{cc} H(q) & ∼\frac{\kappa_{TTS-MF}D_{0}}{\lambda_{TTS-MF}}-1+\sum_{n=0}^{\infty }\frac{\alpha_{TTS}}{\lambda_{TTS-MF}}(1-\frac{\alpha_{TTS}+\kappa_{TTS-MF}D_{0}}{\lambda_{TTS-MF}\left(1+{\tilde{H}}_{fBm}\right)}{)}^{n}\\ H(q) & =\sum_{n=0}^{N}c_{n}\left(q\right)(\frac{\alpha_{TTS}}{\lambda_{TTS-MF}}{)}^{n}+R_{N}\left(\alpha_{TTS}\right). \end{array}$$ (43)

where *N* corresponds to truncation up to the *N*-th order and *R*_*N*_(*α*_*TTS*_) is a residual term or approximation error. Also, note that to guarantee convergence of Eq (43) it suffices that |*α*_*TTS*_ − λ_*TTS*−*MF*_(*H*_*fBm*_(*q*, *ε*) + 1) + *κ*_*TTS*−*MF*_*D*_0_| < 1, as happens for a fractional Brownian motion (see Eq (31) with *H* = *H*_*fBm*_(*q*, *ϵ*)), and hence the name of the approximation made.

Therefore, for simplicity, henceforth assume that

$$\begin{array}{c} H\left(q\right)=\sum_{n=0}^{W}b_{n}\left(q\right)\alpha_{TTS}^{n}, \end{array}$$ (44)

where *W* is the degree of a polynomial of fit and ${\left\{b_{n}\left(q\right)=c_{n}\left(q\right)\lambda_{TTS-MF}^{-n}\right\}}_{n=1}^{W}$ the associated coefficients. Note that if *W* = 1, then it is possible to rewrite the expression (31) for a fractional Brownian motion as a linear function where $b_{1}\left(q\right)=\lambda_{TTS-MF}^{-1}$ and $b_{0}\left(q\right)=D_{0}\kappa_{TTS-MF}\lambda_{TTS-MF}^{-1}-1$. Thus, the Eq (44) corresponds to considering the effects of multifractal time series as a composition of small local time intervals where the system is considered as monofractal. Now, the empirical data that will be used corresponds to 2 financial time series stated in Table 2.

**Table 2:** Stock market index and currency used to explore the relationship between temporal Theil scaling and generalized Hurst exponent from the generalized Shannon index approach. Dates are placed in the ISO universal date format.

Stock index name	Ticker	Start date	Final date
Dow Jones Industrial Average	⌃DJI	1992-01-03	2023-06-07
Euro to Colombian peso	EURCOP = X	2003-01-02	2023-06-07

### 7.1 Relationship of generalized Hurst exponent and the temporal Theil scaling exponent in empirical time series

The process to follow in this section consists of:

1. Take the closing price on each time series defined on a daily basis denoted as *S*_*t*_.
2. Carry out profiling of the time series filtering the days in which the empirical data have a non-zero return.
3. Estimate the logarithmic returns *L*_*t*_ = ln(*S*_*t*+1_) − ln(*S*_*t*_), and absolute log-returns, and volatilities of the log-returns using Eqs (33) and (34), respectively. Thus, the total time series is tripled.
4. Apply the diffusive algorithm for the original time series, absolute log-returns, and volatilities of the log-return.
5. Calculate the cumulative mean, and the Shannon index in each of the diffusive trajectory time series by adding new data for each time *t*.
6. Perform power law regression based on Eq (24) to find the temporal Theil scaling coefficient *K*_*TTS*_(*t*) and the temporal Theil scaling exponent *α*_*TTS*_(*t*). It is important to mention that in all the iterations of this process, one cost function is estimated on the adjustment of the temporal Theil scaling given by the coefficient of determination $R_{TTS}^{2}$.
7. Estimate the generalized Hurst exponent using a multifractal method such as the MF-DFA method discussed in section 3. Then, to avoid the overestimate of the generalized Hurst exponent in short time series that it has already been shown in [65], a threshold value *T*_*MF*_ is chosen such that *H*(*q*) is calculated if and only if *t* ≥ *T*_*MF*_, where *t* represents the size of the cumulative time series at time *t*.
8. Filter the simulations where $R_{TTS}^{2}$ exceeds a certain threshold value *r*_*TTS*_ ≥ 0 and *t* ≥ *T*_*MF*_. Thus, we have that the selected data is from a time *t* ≥ *T*_*MF*_ which must be chosen appropriately since the diffusive trajectory algorithm is of the order $O(t^{2})$.
9. Perform polynomial regression based on Eq (44) to find the coupling coefficients of the generalized Hurst exponent *H*(*q*) with the temporal Theil scaling exponent *α*_*TTS*_ denoted by ${\left\{b_{n}\left(q\right)\right\}}_{n=1}^{W}$. It is important to mention that in all the iterations of this process, two cost function is estimated on the adjustment of Eq (44) given by the coefficient of determination *R*^2^(*q*) and the *p*-th mean absolute error *MAE*_*p*_(*q*) defined in Eq (35).
10. Repeat the above process with different values of *q*, say $\tilde{W}$ values, i.e. with $q\in {\left\{q_{k}\right\}}_{k=1}^{\tilde{W}}$.

Finally, before going on to the results after having done this process, it is worth mentioning that the parameters selected were *r*_*TTS*_ = 0.95, *T*_*MF*_ = 4096, *W* = 4, $\tilde{W}=4$, *q* ∈ {−2, −1, 1, 2}. Regarding the estimation of the generalized Hurst exponent with the MF-DFA method, a library built in Python is used to make this calculation quite efficient [61]. In addition, all the codes made are published in the Github [67]. At last, to obtain the uncertainty in the parameters estimated by the least squares regression in some adjustments, the covariance matrix *Cov*(⋅) is calculated with the vector of residuals of the fit $\vec{e}$, that is, $Cov\left(\cdot \right)\propto \vec{e}{\vec{e}}^{T}$. Then, the square root of the respective term on the diagonal of the covariance matrix is taken as the error in the fit parameters.

**Fig 2:**
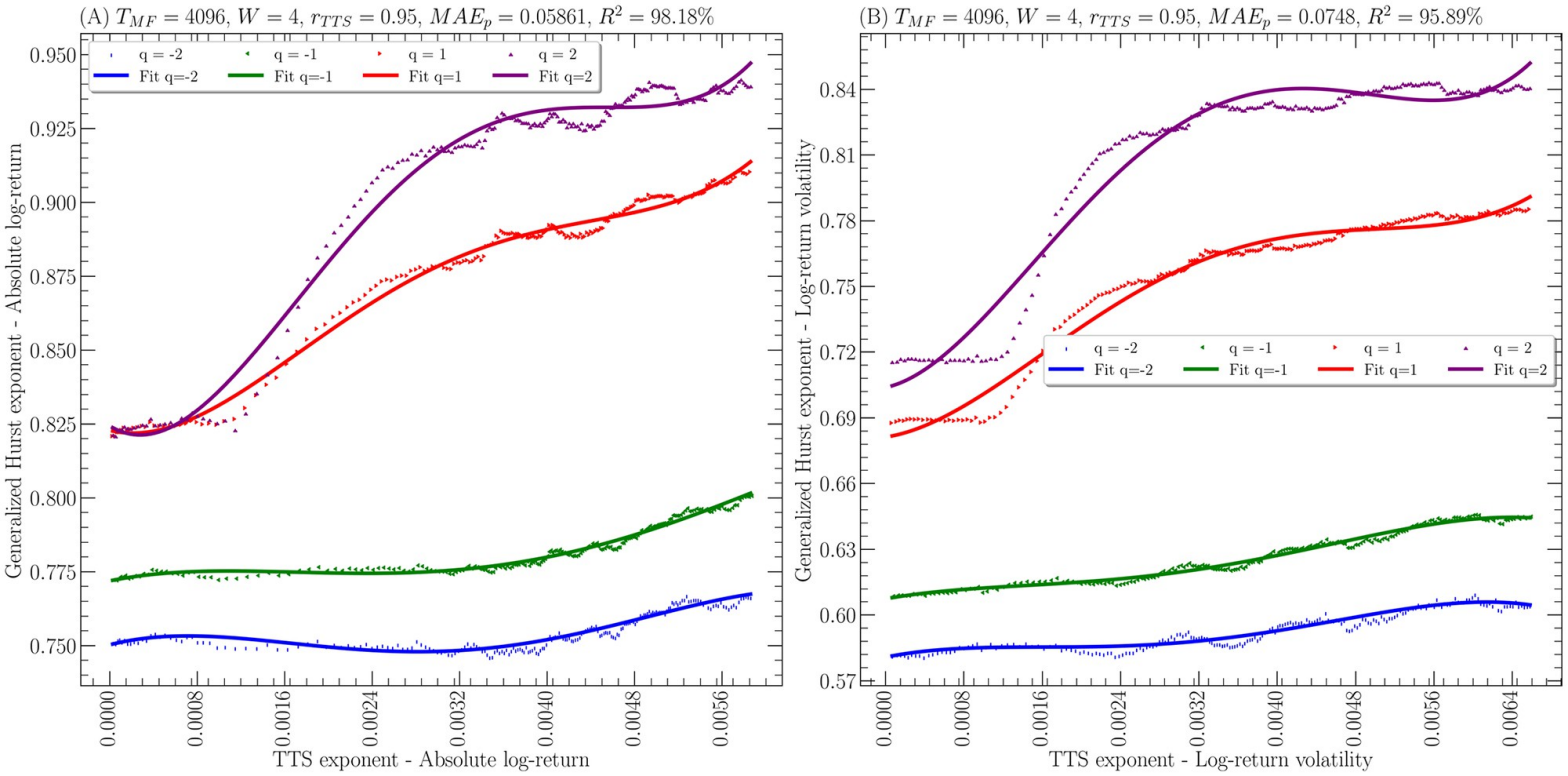
Generalized Hurst exponent *H*(*q*) as a function of temporal Theil scaling exponent *α*_*TTS*_(*t*) for the time series of the Dow Jones Industrial Average measured daily from January 3, 1992, to June 7, 2023. **(A)** absolute log-returns time series. **(B)** volatilities of the log-returns time series. In all cases, the empirical data is shown as points and the theoretical fitting to (44) with *W* = 4 as a line.

**Fig 3:**
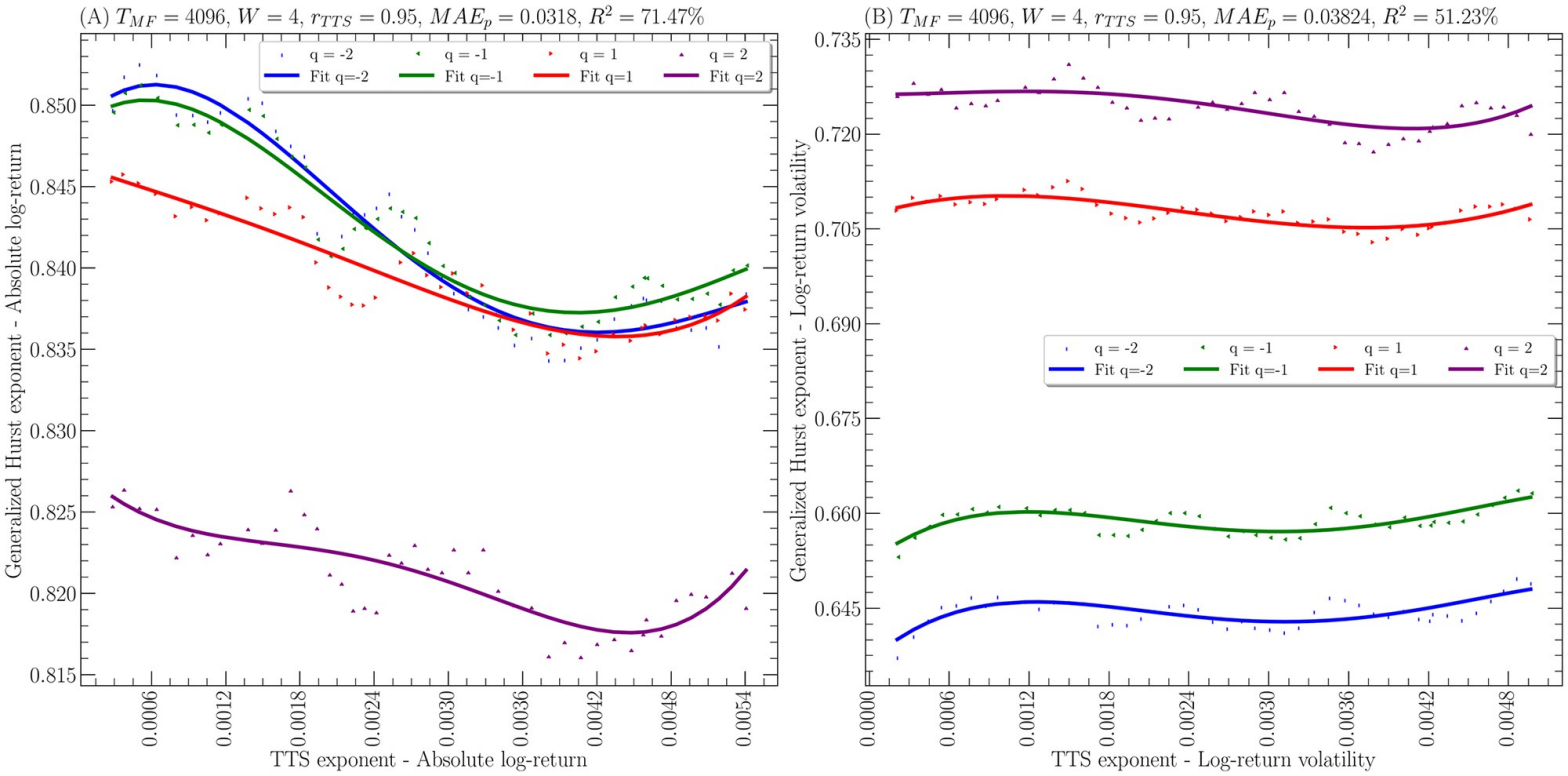
Generalized Hurst exponent *H*(*q*) as a function of temporal Theil scaling exponent *α*_*TTS*_(*t*) for the time series of the Euro to Colombian peso currency measured daily from January 2, 2003, to June 7, 2023. **(A)** absolute log-returns time series. **(B)** volatilities of the log-returns time series. In all cases, the empirical data is shown as points and the theoretical fitting to (44) with *W* = 4 as a line.

Figs 2 and 3 show the generalized Hurst exponent as a function of the temporal Theil scaling exponent for the given parameters. Hence, it is clear that in all cases the polynomial fit works quite well to capture the fluctuations of the generalized Hurst exponent as a function of the temporal Theil scaling exponent. Specifically, the points represent the empirical data obtained on the 2 financial time series while the solid line represents the polynomial fit for the different values of *q*. Indeed, the smallest coefficient of determination *R*^2^(*q*) is 49.56% for the *EURCOP = X* with *q* = −2. Also, it is worth noting that the scales of all the figures respect that the generalized Hurst exponent is about 2 orders of magnitude larger than the temporal Theil scaling exponent. It is important to note that regardless of the case, the range covered by the temporal Theil scaling exponent is of the order of 10^−4^ ∼ 10^−3^. Furthermore, Table 3 shows the coefficients of determination *R*^2^(*q*) obtained in each case and also shows the highest value obtained from the regression coefficients, that is

$$\begin{array}{c} B\left(q\right)=\underset{1\leq n\leq W}{\text{max}}\left\{|b_{n}\left(q\right)|\right\}. \end{array}$$ (45)

**Table 3:** Parameters of the relationship between the generalized Hurst exponent *H*(*q*) and the temporal Theil scaling exponent *α*_*TTS*_ obtained after the fit with the expression (36). Note that for currency ticker, = *X* is removed in its name.

Symbol	Time Series	*q*	*R*^2^(*q*)(%)	*MAE*_1_(*q*)	B(q)
⌃DJI	Absolute Log-Return	-2	91.86	0.03559	1.87 × 10^8^ ± 1.58 × 10^6^
⌃DJI	Absolute Log-Return	-1	97.45	0.03089	8.78 × 10^7^ ± 1.21 × 10^6^
⌃DJI	Absolute Log-Return	1	98.98	0.04278	5.01 × 10^8^ ± 2.45 × 10^6^
⌃DJI	Absolute Log-Return	2	98.18	0.05861	9.87 × 10^8^ ± 4.80 × 10^6^
⌃DJI	Log-Return Volatility	-2	93.15	0.03913	1.43 × 10^8^ ± 1.29 × 10^6^
⌃DJI	Log-Return Volatility	-1	98.21	0.03430	1.10 × 10^8^ ± 9.31 × 10^5^
⌃DJI	Log-Return Volatility	1	97.71	0.05565	3.60 × 10^8^ ± 2.76 × 10^6^
⌃DJI	Log-Return Volatility	2	95.89	0.07480	6.77 × 10^8^ ± 5.09 × 10^6^
EURCOP	Absolute Log-Return	-2	93.11	0.03329	1.32 × 10^8^ ± 9.90 × 10^6^
EURCOP	Absolute Log-Return	-1	91.77	0.03153	1.11 × 10^8^ ± 9.10 × 10^6^
EURCOP	Absolute Log-Return	1	87.53	0.02804	3.77 × 10^7^ ± 7.69 × 10^6^
EURCOP	Absolute Log-Return	2	71.47	0.03180	1.58 × 10^8^ ± 9.98 × 10^6^
EURCOP	Log-Return Volatility	-2	49.56	0.03394	2.13 × 10^8^ ± 1.43 × 10^7^
EURCOP	Log-Return Volatility	-1	54.8	0.03133	2.01 × 10^8^ ± 1.25 × 10^7^
EURCOP	Log-Return Volatility	1	65.16	0.03033	1.98 × 10^7^ ± 1.15 × 10^7^
EURCOP	Log-Return Volatility	2	51.23	0.03824	1.39 × 10^8^ ± 1.92 × 10^7^

Hence, in Table 3 all the values *B*(*q*) are in the same order of magnitude around 10^8^ and coincide with the coefficient of the polynomial with the highest degree, that is, $\alpha_{TTS}^{W}$, which implies that the contribution of this term is really of the order of 10^8−4*W*^ ∼ 10^8−3*W*^, that is, 10^−8^ ∼ 10^−4^.

Finally, it is worth mentioning that in Figs 2 and 3, it is observed that the relationship between the generalized Hurst exponent and the temporal Theil scaling exponent is not a bijection between these two quantities due to the non-monotonic behavior of the curves, which becomes clearer when remembering the polynomial relationship of Eq (44). Nevertheless, it is important to remember that the LA-fBm is a local approximation, which implies that this non-monotonic behavior must be interpreted in terms of the time series having a multifractal behavior presenting moments of smaller or greater long-range correlation even when the temporal Theil scaling exponent increases.

### 7.2 Optimal selection of moments for the calculation of the generalized Hurst exponent

Now, noting the high orders of magnitude of these fit parameters in Table 3, it can be thought that *b*_*n*_(*q*) is associated with a measure of the most optimal value $\tilde{q}$ with which to calculate the generalized Hurst exponent in a time series. To do this, from now on we set (for simplicity) $\tilde{W}=W+1$, and observe that Eq (44) generates the following system of equations

$$\begin{array}{c} \underset{H}{\underset{︸}{\left(\begin{array}{c} H(q_{1})\\ H(q_{2})\\ \vdots \\ H(q_{W+1}) \end{array}\right)}}=\underset{A}{\underset{︸}{\left(\begin{array}{cccc} b_{0}\left(q_{1}\right) & b_{1}\left(q_{1}\right) & \cdots & b_{W}\left(q_{1}\right)\\ b_{0}\left(q_{2}\right) & b_{1}\left(q_{2}\right) & \cdots & b_{W}\left(q_{2}\right)\\ \vdots & \vdots & \ddots & \vdots \\ b_{0}\left(q_{W+1}\right) & b_{1}\left(q_{W+1}\right) & \cdots & b_{W}\left(q_{W+1}\right) \end{array}\right)}}(\begin{array}{c} \alpha_{TTS}^{0}\\ \alpha_{TTS}^{1}\\ \vdots \\ \alpha_{TTS}^{W} \end{array})\\ H(q_{1},q_{2},\ldots ,q_{W+1})=A\left(q_{1},q_{2},\ldots ,q_{W+1}\right){\vec{\alpha }}_{TTS}. \end{array}$$ (46)

Therefore, it is worth remembering that the spectral norm of $A(q_{1},\ldots ,q_{W+1})$ evaluates the square root of the largest eigenvalue of the matrix $A^{T}A$. This computation provides an approximation of the most significant eigenvector in the matrix, denoted as $\vec{z}=(z\left(q_{1}\right),z\left(q_{2}\right),\ldots ,z\left(q_{W+1}\right))$. Consequently, selecting the largest absolute component of $\vec{z}$ enables the estimation of $\tilde{q}\in q_{1},\ldots ,q_{W+1}$, which contributes the most to the computation of the generalized Hurst exponent given the temporal Theil scaling exponent *α*_*TTS*_(*t*). In simpler terms, the spectral norm of $A(q_{1},\ldots ,q_{W+1})$ and its corresponding eigenvector furnish an algorithm for identifying the optimal *q*-th moment of the probability distribution associated with an empirical time series to enhance the accuracy of generalized Hurst exponent estimation given the temporal Theil scaling exponent *α*_*TTS*_(*t*). In fact, it is important to remember that precisely one of the sources of multifractality in financial time series is the probability distribution associated with said time series rather than the temporal correlations between different time periods that can be destroyed by reordering randomly the time series as shown in [68].

It’s important to emphasize that the *q*-th optimal moment algorithm is inapplicable to fractional Brownian motion. In the case of a monofractal system like fractional Brownian motion, selecting any arbitrary value for *q* would be sufficient to obtain the Hurst exponent *H*. In this context, constructing the matrix A from the expression (46) and considering Eq (31) results in a matrix of size 2 × 1, as only one *q* value and two coefficients are necessary for the linear regression (see Eq (31)). Consequently, the spectral norm would be computed for a 1 × 1 matrix, which is trivial for the *q*-th optimal moment selection algorithm. As a result, this approach would not reveal any significant difference in the system’s behavior, unlike the two financial time series described in Table 2 as shown below.

Now, the matrix A has been computed for each financial time series outlined in Table 2. Subsequently, Table 4 displays the spectral norm [69] alongside the associated eigenvector components. Indeed, the optimal value $\tilde{q}\in -2,-1,1,2$ for ⌃*DJI* and *EURCOP = X* is determined as 1 and −2 for absolute log-returns and volatilities of log-returns, respectively. Specifically, in the case of *EURCOP = X*, components corresponding to *q* = −2 and *q* = 1 exhibit slight differences. This observation suggests that, for the time series *EURCOP = X*, minor fluctuations play a more crucial role in the system’s behavior, whereas for ⌃*DJI*, larger fluctuations hold greater significance. This inference aligns with the known stability of *EURCOP = X* as a currency over the presented time period in Table 2, signifying its frequent occurrence of both positive and negative returns with relatively small values. Similarly, ⌃*DJI*, being a stock index that has experienced consistent growth during the study period in Table 2, reflects predominantly positive returns, with occasional minor negative returns compensated by substantial increases in the stock index’s value.

**Table 4:** Spectral norm and eigenvector associated to the matrix A used to estimate the optimal value $\tilde{q}$ for each of the 2 financial time series defined in the Table 2 according to Eq (46). Note that for currency tickers, = *X* is removed in its name.

Symbol	Time series	q	Spectral norm	Eigenvector component
⌃DJI	Absolute Log-Return	-2	1.126 × 10^9^	-0.1662
⌃DJI	Absolute Log-Return	-1	1.126 × 10^9^	-0.3625
⌃DJI	Absolute Log-Return	1	1.126 × 10^9^	0.7762
⌃DJI	Absolute Log-Return	2	1.126 × 10^9^	0.4883
⌃DJI	Log-Return Volatility	-2	7.881 × 10^8^	-0.1811
⌃DJI	Log-Return Volatility	-1	7.881 × 10^8^	-0.4643
⌃DJI	Log-Return Volatility	1	7.881 × 10^8^	-0.6983
⌃DJI	Log-Return Volatility	2	7.881 × 10^8^	-0.5138
EURCOP	Absolute Log-Return	-2	2.373 × 10^8^	0.5573
EURCOP	Absolute Log-Return	-1	2.373 × 10^8^	-0.5013
EURCOP	Absolute Log-Return	1	2.373 × 10^8^	-0.5552
EURCOP	Absolute Log-Return	2	2.373 × 10^8^	-0.3604
EURCOP	Log-Return Volatility	-2	3.248 × 10^8^	-0.6565
EURCOP	Log-Return Volatility	-1	3.248 × 10^8^	0.2566
EURCOP	Log-Return Volatility	1	3.248 × 10^8^	0.6166
EURCOP	Log-Return Volatility	2	3.248 × 10^8^	-0.3508

Lastly, for the sake of comparing this method of selecting the *q*-th optimal moment value with other proposed methodologies, such as the MF-DFA method, it is noteworthy that in computing the generalized Hurst exponent using MF-DFA, it is customary to identify the *q*-th optimal moment where the overall detrended fluctuation functions $F\left(q,\delta \right)∼\delta^{H(q)}$ and $F\left(q+1,\delta \right)∼\delta^{H(q+1)}$ display significant similarity [31, 61]. Consequently, if the *q*-th moment is varied continuously, this convergence typically occurs (as a first approximation) whenever $\frac{db_{n}\left(q\right)}{dq}=0$, for all $n\in \{1,2,\ldots ,\tilde{W}\}$, since from Eq (44) it follows that

$$\begin{array}{c} \|F(q+\eta ,\delta )-F(q,\delta )\|∼\|\delta^{H(q+\eta )}-\delta^{H(q)}\|\approx \eta \delta \;\text{ln}(\delta )\|\frac{dH(q)}{d\eta }\|∼\left|\sum_{n=0}^{\tilde{W}}\alpha_{TTS}^{n}\frac{db_{n}\left(q\right)}{dq}\right|, \end{array}$$ (47)

for all *η* > 0. Hence, the optimal *q*-th moment value in the MF-DFA method is chosen as the one where the generalized Hurst exponent as a function of the temporal Theil scaling exponent does not vary much in its parameters $\{b_{n}\left(q\right){\}}_{n=1}^{\tilde{W}}$. Nonetheless, this approach may introduce bias into the matrix A by generating two rows with closely resembling values, making it challenging to discern the most optimal *q*-th moment value. Therefore, it is crucial to emphasize that the selection of the *q*-th optimal moment presented in this study involves identifying the most representative *q*-th moment value during the computation of the generalized Hurst exponent across a range of values $q_{1},q_{2},\ldots ,q_{\tilde{W}}$. This selection process aims to capture the most significant fluctuations within a time series, whether positive or negative, as shown with the series of time of ⌃*DJI* and *EURCOP = X*.

## 8 Conclusions

In summary, we establish a theoretical link between the Hurst exponent *H* and the temporal Theil scaling exponent *α*_*TTS*_ through the multifractality partition function approach. Specifically, Section 4 introduces the generalized Shannon index S(q), extending the Shannon index, while subsection 5 defines the partition function Ω_*TTS*_(*q*, *δ*) and the multifractal exponent of the generalized Shannon index *β*_*TTS*_(*q*) that relates with the generalized Hurst exponent *H*(*q*). For a fractional Brownian motion, this exponent *β*_*TTS*_(*q*) is expressed in Eq (31). Also, in Section 6, we validate the *α*_*TTS*_ and *H* relationship for fractional Brownian motion. Multiple simulations with 512 time steps show a linear regression with positive λ_*TTS*−*MF*_ and negative *κ*_*TTS*−*MF*_ coupling constants. The *R*^2^ is 84.96% for volatilities of log-returns, indicating a high-quality fit.

Finally, Section 7 applies the relationship between the temporal Theil scaling exponent *α*_*TTS*_ and the generalized Hurst exponent *H*(*q*) to construct a selection algorithm for the optimal *q*-th moment, obtaining $\tilde{q}=1$ and $\tilde{q}=-2$ values for ⌃*DJI* and *EURCOP = X*, respectively. The algorithm suggests that small fluctuations are more relevant for *EURCOP = X*, while large fluctuations dominate the behavior of ⌃*DJI*. All results are accessible on Github [67].

From a practical standpoint, it’s crucial to note that while the findings in this article pertain to two specific financial time series, the algorithm for determining the optimal q-th moment may be applied to other financial datasets. This addresses two common challenges associated with employing the Hurst exponent in financial time series analysis. Firstly, it resolves the issue of selecting the most suitable q-th moment for estimating the generalized Hurst exponent. Secondly, it provides a solution for calculating the Hurst exponent in scenarios where data distribution is limited, i.e., when dealing with short time series. This is particularly significant as the relationship between *α*_*TTS*_ and *H*(*q*) (see Eq (44)) implies that the Generalized Hurst exponent could be estimated by knowing the temporal Theil scaling exponent, not only in the case of a fractional Brownian motion. Also, with respect to this second problem, it is worth mentioning that there are other methods to estimate the Hurst exponent in short time series, for example, by maximum likelihood which has been verified in a fractional Brownian motion and an extension of this stochastic process known as fractional Brownian bridge [70]. Nevertheless, since the comparison of the Hurst exponent in short time series using different methods is beyond the scope of this article, it is proposed as a future direction of work.
